# Transcript profiling of hairy vetch (*Vicia villosa* Roth) identified interesting genes for seed dormancy

**DOI:** 10.1002/tpg2.20330

**Published:** 2023-05-01

**Authors:** Shahjahan Ali, Lisa Kissing Kucek, Heathcliffe Riday, Nick Krom, Sarah Krogman, Kimberly Cooper, Lynne Jacobs, Perdeep Mehta, Michael Trammell, Suresh Bhamidimarri, Twain Butler, Malay C. Saha, Maria J. Monteros

**Affiliations:** ^1^ USDA‐ARS, US Dairy Forage Research Center Madison Wisconsin USA; ^2^ Noble Research Institute, LLC Ardmore Oklahoma USA; ^3^ Oklahoma State University Cooperative Extension Shawnee Oklahoma USA; ^4^ Corteva Agriscience Connell Washington USA; ^5^ Bayer Crop Science Chesterfield Missouri USA

## Abstract

Hairy vetch, a diploid annual legume species, has a robust growth habit, high biomass yield, and winter hardy characteristics. Seed hardness is a major constraint for growing hairy vetch commercially. Hard seeded cultivars are valuable as forages, whereas soft seeded and shatter resistant cultivars have advantages for their use as a cover crop. Transcript analysis of hairy vetch was performed to understand the genetic mechanisms associated with important hairy vetch traits. RNA was extracted from leaves, flowers, immature pods, seed coats, and cotyledons of contrasting soft and hard seeded “AU Merit” plants. A range of 31.22–79.18 Gb RNA sequence data per tissue sample were generated with estimated coverage of 1040–2639*×*. RNA sequence assembly and mapping of the contigs against the *Medicago truncatula* (V4.0) genome identified 76,422 gene transcripts. A total of 24,254 transcripts were constitutively expressed in hairy vetch tissues. Key genes, such as *KNOX4* (a class II KNOTTED‐like homeobox KNOXII gene), *qHs1* (endo‐1,4‐β‐glucanase), *GmHs1‐1* (calcineurin‐like metallophosphoesterase), chitinase, shatterproof 1 and 2 *(SHP1, SHP2)*, shatter resistant 1–5 *(SHAT1–5)*(NAC transcription factor), *PDH1* (prephenate dehydrogenase 1), and pectin methylesterases with a potential role in seed hardness and pod shattering, were further explored based on genes involved in seed hardness from other species to query the hairy vetch transcriptome data. Identification of interesting candidate genes in hairy vetch can facilitate the development of improved cultivars with desirable seed characteristics for use as a forage and as a cover crop.

## Core Ideas

- Transcriptome sequencing of hairy vetch tissues identified 76,422 genes with 24,254 genes expressed constitutively.
- Key candidate genes involved in seed harness, pod shattering, and other agronomic traits have been identified.
- Transcript sequence information will be useful in annotating hairy vetch genomes.
- The resources we generated will be useful in developing soft seeded hairy vetch cultivars.

**Abbreviations**
*ALC*
: ALCATRAZ gene
*AP2*
: APETALA2
*Asp*
: ASPER gene
BSA: bulk segregation analysis
BSR‐Seq: bulked segregant RNA Seq
CT: cycle threshold
CV: coefficient of variation
FPKM: fragments per kilobase of transcript per million
*Gmhs1‐1*
: calcineurin‐like metallo‐phosphoesterase
*IND*
: INDEHISCENT gene
*KNOX4*
: *class II KNOTTED‐like homeobox KNOXII* gene
MACE: massive amplification of cDNA ends
*PDH1*
: prephenate dehydrogenase 1
*qHS1*
: endo‐1,4‐β‐glucanase
RIN: RNA integrity number
*SHAT1–5*
: *shatter resistant* 1*–*5
*SHP1 and SHP*
: SHATTERPROOF 1 and 2
*SRG*
: STAY‐GREEN

## 1 INTRODUCTION

Hairy vetch (*Vicia villosa* Roth) is a cool season legume with broad adaptability, winter hardiness, and vigorous growth potential (Mirsky et al., 2017; Parr et al., 2011). As a legume, hairy vetch has the capacity for symbiotic nitrogen fixation. Hairy vetch biomass contributes an estimated equivalent of 100–230 kg ha^−1^ N when used as a cover crop (Grossman et al., 2018). It is an annual, diploid, and allogamous plant species (Kahlaoui et al., 2009) with a base chromosome number of 2*n* = 2*x* = 14 and a predicted genome size of 2000 Mb (Macas et al., 2015).

Despite impressive performance, hairy vetch is not readily adapted by grain farmers due to the species’ lack of domestication. *V. villosa* suffers from seed hardness, which was eliminated in most crop species thousands of years ago (Doebley et al., 2006; Pickersgill, 2007; Purugganan & Fuller, 2009; Smýkal et al., 2014). Removal of seed dormancy and pod shattering were the two most important traits in legume domestication (Smýkal et al., 2014).

Seed hardness often termed synonymously seed dormancy. Physiological seed dormancy is commonly caused by inhibiting chemicals inside the seeds especially interactions between abscisic acid and gibberellins to induce and maintain dormancy (Finch‐Savage & Leubner‐Metzger, 2006, Soltani et al., 2021). Seed hardness is a physical characteristic of the seed that prevents uptake of water and promotes longer dormancy. Impermeable seeds often have a hard seed coat characterized by pectinaceous outer layer of palisade cells, higher lignin content, and fatty acid composition of the cutin layer (Kannenberg & Allard, 1964; Shao et al., 2007; Werker et al., 1979). Seed hardness in hairy vetch is a target for improvement in breeding programs aimed at specific commercial markets. Hard seeded cultivars are useful in some self‐reseeding forages systems, whereas the soft seeded cultivars are highly desirable for use in cover crop systems. Hairy vetch plants with hard seed and some degree of pod shattering can grow hairy vetch seed in the soil seedbank thus reducing the need for and associated costs of reseeding this annual species. However, shattered seeds contribute to seed losses during seed production and emerging plants can become weeds to the future crops in crop rotations (Mirsky et al., 2015). Further, cultivars with hard seed can lead to uneven strand establishment due to variable germination rates and seedling emergence.

Seed hardness in hairy vetch displays genetic variation but also influenced by local growth environment (Jacobsen et al., 2010; Kissing Kucek et al., 2020; Mirsky et al., 2015; Renzi et al., 2016). Factors affecting seed hardness include plant genetic variation and external factors such as relative humidity, pod maturity, and postharvest processing of the pods. Exposure of hairy vetch seeds to high temperature during storage results in the degradation of the palisade tissues, lipids, and hydrophobic compounds of the seed coat affecting the seed hardness (Renzi et al., 2014).

Understanding the genetic mechanisms underlying soft versus hard seed development in hairy vetch can facilitate breeding applications aimed at developing enhanced hairy vetch cultivars for dual purpose applications. Seed dormancy in legumes is affected by only a few loci. A single gene regulates fall dormancy in lentil (*Lens culinaris* Medic) (Ladizinsky, 1985), lupin (*Lupinus angustifolius* L.) (Forbes & Wells, 1968), cowpea (*Vigna unguiculata* L.) (Kongjaimun et al., 2012), ricebean (*Vigna umbellata* (Thunb.) Ohwi and Ohashi) (Isemura et al., 2012), and *Medicago truncatula* Gaertn (Chai et al., 2016). Two or three genes are associated with seed dormancy in pea (*Pisum sativum*) (Weeden, 2007). In soybean (*Glycine max*), the *Gmhs1‐1* gene mediates seed dormancy by modifying calcium related protein in the seed coat membrane protein (Sun et al., 2015). The *qHS1* mutant in soybean has increased water imbibition due to changes in the seed coat (Jang et al., 2015; Sun et al., 2015). In common vetch (*Vicia sativa* L.), at least two loci are associated with seed hardness with dominance at A locus resulted into hard seed, whereas recessive homozygous (aa) and dominance at B locus produce soft seed (Donnelly et al., 1972). In *M. truncatula*, Chai et al. (2016) described a breakdown of physical dormancy in *KNOX4*, a class II KNOTTED‐like homeobox (*KNOXII*) mutant line. In black bean (*Phaseolus vulgaris*), *ASPER (Asp)* controls seed hardness and expressed in seed coat luster (Bassett, 1996; Bushey et al., 2000). Genotypes with mutant *Asp (asp/asp)* gene displayed dull/matte/opaque seed coats, whereas seed coat shininess has been associated with a low rate of water uptake in genotypes (Bushey et al., 2000).

**Core Ideas**

- Transcriptome sequencing of hairy vetch tissues identified 76,422 genes with 24,254 genes expressed constitutively.
- Key candidate genes involved in seed harness, pod shattering, and other agronomic traits have been identified.
- Transcript sequence information will be useful in annotating hairy vetch genomes.
- The resources we generated will be useful in developing soft seeded hairy vetch cultivars.

Hairy vetch is an outcrossing species, and released varieties are a composite/synthetic of diverse genotypes. Thus, the traits are not genetically fixed in any of the hairy vetch cultivars. Heritability studies of hard seed in this species were performed extensively by screening over 3000 genotypes in diverse environments revealed that seed hardness is a highly heritable trait (Kissing Kucek et al., 2020). It is common for a plant of vetch to produce both hard and soft seeds. We have screened over 5000 plants in the breeding program and have seen this pattern. There may not be a single gene responsible for the trait that can be fixed.

We used an approach similar to BSA (bulk segregation analysis) to separate soft and hard seeds after germination tests and by employing sulfuric acid scarification technique to ungerminated or hard seeds. BSA refers to the process of pooling samples based on similar phenotypes such as disease susceptible versus resistance or drought tolerant versus susceptible, but arbitrary for other traits. BSA method was used in generating resistance and susceptible pools of downy mildew of lettuce. Each pool contains individuals identical for genomic regions for resistance or susceptible trait but arbitrary at all unlinked regions (Michelmore et al., 1991). The authors identified three random amplified polymorphic DNA markers in lettuce linked to a gene for resistance to downy mildew. They also concluded that BSA has several advantages over the use of near‐isogenic lines to identify markers in specific regions of the genome. BSR‐Seq (bulked segregant RNA Seq) was performed on two pools of maize segregating for gl3 locus, a recessive mutant gene involved in the accumulation of epicuticular wax (Liu et al., 2012), and it was revealed that this method efficiently mapped genes even in populations for which no polymorphic markers had been identified previously. Miller et al. (2013) separated *N*‐ethyl‐*N*‐nitrosourea‐induced mutants and wild‐type genotypes in zebrafish following BSA method. They observed that the combination of BSA and RNA Seq was powerful way in mapping known and unknown mutants to unique regions of the genome in tetraploid wheat BSA followed by RNA sequencing was found very effective in identifying SNPs in genic region of grain protein content (GPC) gene, GPC‐B1 (Zou et al., 2016). BSA techniques followed by RNA sequencing have been used in multiple plant species such as maize, wheat, red clover, and melon for differential gene expression and SNP discovery between bulked pools of samples (Chayut et al., 2015; Du et al., 2017; Liu et al., 2012; Trick et al., 2012; Yates et al., 2014). Recently, BSA technique has been used to separate hard and soft seeds of common bean for QTL mapping of water imbibing traits (Soltani et al., 2021).

Genomic resources of hairy vetch are currently limited to a small set of nucleotide sequences (583) available in the NCBI database as of April 20, 2022 (https://www.ncbi.nlm.nih.gov/nuccore//?term=hairy±vetch) and a unpublished draft assembly of the genome, Vvil1.0 (personal communication, Heathcliffe Riday, USDA‐ARS, Madison, WI, USA). Strategies to develop genomic resources for hairy vetch, including transcriptome sequences, can inform genome annotation, provide a framework for tissue‐specific gene expression atlases, and serve as a building block to understand mechanisms associated with seed hardness and other traits with agricultural relevance and to infer breeding decisions. mRNA sequencing in next generation sequencing platform has shown unprecedented complexity of the transcriptomes, provides opportunities to identify genes or genes of interest, gene expression levels, developing functional molecular markers, and enables comparative genomic analysis.

The objectives of this research are to identify the genes expressed in hairy vetch tissues, explore tissue‐specific differential gene expressions, and identify genes associated with important traits in this versatile legume species.

## 2 MATERIALS AND METHODS

### 2.1 Plant materials

One hundred seeds from hairy vetch cultivar, “AU Merit,” were screened for hard or soft seeds. We received AU Merit seeds from Auburn University, Auburn, AL, where it was bred for high forage yield. The environmental coinditions of the growth period were unknown to us. Seeds were stored at 4°C until imbibation. Sixty‐seven seeds that imbibed water as indicated by the swelling of the seeds and germination were referred to as soft. Soaking of the seeds was carried out at room temperature, and germination test was performed by spreading seeds on wet blotting paper placed in Petri dish and incubated for a week at growth room having temperature of 30°C. Thirty‐three seeds that did not imbibe water were scarified by submerging seeds in 100% sulfuric acid (Sigma‐Aldrich, Inc., St. Louis, MO, USA) for 2 min, followed by neutralization with 1 M sodium bicarbonate solution (Sigma‐Aldrich, Inc., St. Louis, MO, USA) and washed thoroughly with Milli‐Q (Milli‐Q Direct Water Purification System, MilliporeSigma, Burlington, MA, USA) water. Twenty‐five seeds that germinated after acid scarification were labeled as hard seed. It took another two rounds of scarification and germination test to make the germination of the remaining eight seeds, and they were not included in this experiment. Both soft and hard seeded plants were grown in a greenhouse with average temperature of 23°C, and 16–8 h day–night settings with maximum light brightness of 893 uE. The minimum and maximum temperatures were recorded as 16 and 31.3°C, respectively, throughout the growth period. Fifteen plants were randomly selected from each of the soft and hard categories. Plants were arranged in randomized block design with three replications. Ten plants (five from hard and five from soft seeds) were placed in two rows in a block. Plants were randomized within rows (3 blocks × 2 rows × 5 plants in a row = 30). Tissues were harvested from leaves, flowers, immature pods, seed coats, and cotyledons (seed without seed coat) from individual plants and were immediately frozen in liquid nitrogen and stored at −80°C until use. To minimize the variations due to the physiological developmental stage, leaves were harvested at 25 days after germination. Similarly, flowers were harvested when plants produced 4–5 racemes prior to anthesis. Pollination between soft or hard seeded plants was performed by bumblebees (genus: *Bombus*) in separate batches to avoid crossing between soft and hard seeded plants. Five immature pods were harvested from each plant 20 days after pollination. Seed coats were peeled off from 15 to 20 physiologically mature seeds indicated by dark brown seed coat color that also enabled easily peeling of the seed coats manually. Cotyledons underneath the seed coat constituted the cotyledon samples. Total RNA was isolated from individual plants (described in following section), and then equal amount of RNA from each plant within a replication was pooled to make a biological replication for soft or for hard seeded plants. Similar approach was used for pooling RNA samples of alfalfa from each replicate in alfalfa RNA Seq experiments (Yang et al., 2011).

### 2.2 RNA isolation, quantitation, and pooling

Total RNA from individual plants was isolated using PureLink RNA Mini Kit (Invitrogen, Carlsbad, CA, USA) according to the manufacturer's instructions with some modifications. These were targeted tailored to isolating RNA from immature pods, seed coat, and cotyledons (seeds without seed coat). Briefly, for the leaves and flowers, 100–200 mg of tissue was placed into a chilled 2.0 mL safe‐lock microfuge tube and combined with three (2.5 mm diameter) glass beads (BioSpec Inc., Bartlesville, OK, USA). Tubes were then placed in a chilled metal rack, and tissues were grounded into fine powder using in Mini‐BeadBeater (BioSpec Inc., Bartlesville, OK, USA) for 1 min and 45 s. Cold 1 mL lysis buffer containing 1% 2‐mercaptoethanol (Sigma‐Aldrich, Inc., St. Louis, MO, USA) was added to the sample, vortexed vigorously, and incubated at room temperature for 5 min. The samples were then centrifuged (Eppendorf Centrifuge 5425 R, Enfield, CT, USA) at 12,000 g for 5 min. Clear supernatant was transferred into a new RNase‐free 2.0 mL tube, added half volume of absolute ethanol, and loaded into mini columns. After washing the column with 350 μL wash buffer 1, 80 μL of DNase solution (0.4 U/μL) (Invitrogen, Carlsbad, CA, USA) was added into the column, incubated at room temperature for 30 min, and the columns were washed with 350 μL wash buffer 1. Columns were further washed twice with 500 μL wash buffer 2 followed by a dry spin of 1 min. RNAs from the columns were eluted with 100 μL RNase‐free water.

Pod, seed coat, and cotyledon samples were grounded with mortar and pestle using liquid nitrogen. A total of 25–50 and 50–100 mg of ground tissues were used for seed coat and pods tissues, respectively. The following modifications were also introduced in the protocol: 2% PVP4000 and 10% 2‐mercaptonol (Sigma‐Aldrich, Inc., St. Louis, MO, USA) were added in the lysis buffer. After the incubation of samples in lysis buffer and centrifugation as mentioned above, half volume of 3 M KAc (potassium acetate), pH 6.8 was added in the clear supernatant, incubated on ice for 30 min, and centrifuged (Eppendorf Centrifuge 5425 R, Enfield, CT, USA) at 12,000 g for 30 min. Then remaining steps of RNA mini kit protocol were followed. The elution volume for pod RNA was 100 μL and for seed coat was 30 μL.

RNA extraction for cotyledon was performed using Trizol reagent (Invitrogen, Carlsbad, CA, USA). A total of 1 mL of Trizol was added to 25 mg of tissue and combined in a 2.0 mL Eppendorf tube. After phase extraction with 200 μL chloroform (Sigma‐Aldrich Ltd, St. Louis, MO, USA), the clear upper phase was transferred into a new 1.5 mL Eppendorf tube. Half volume of absolute ethanol was added to the supernatant, loaded into the column and followed the remaining procedure of RNA mini kit protocol. The cotyledon RNA was eluted with 30 μL RNase‐free water.

RNA concentration for all samples was measured with broad or high sensitivity kits using Qubit 4.0 (Invitrogen, Carlsbad, CA, USA) depending of concentration of RNA of the samples. RNA qualities were tested by measuring RIN (RNA integrity number) using RNA IQ kit and Qubit 4.0.

### 2.3 RNA Seq library preparation, sequencing, and data analysis

mRNA (PolyA+) was isolated from 100 ng of total RNA followed by RNA Seq library preparation using TruSeq Stranded mRNA library preparation kit (Illumina Inc, San Diego, CA, USA). The sequencing runs were performed in HiSeq4000 (Illumina, San Diego, CA, USA) with 2 × 150 bp PE read setting.

The sequence reads of each sample were quality trimmed to remove low quality bases (*Q* score of less than 30), primer/adapter sequences, and reads that were less than 30 bp using the Trimmomatic software (v 0.36) (Bolger & Giorgi, 2014). A single super‐assembly of all the sample reads was performed in Trinity (v 2.8.3) (Grabherr et al., 2011). Contigs were then mapped to the *M. truncatula* genome (v 4.0) with HISAT2 version 2.0.5 (https://ccb.jhu.edu/software/hisat2/index.shtml) using the default mapping parameters. Transcript expression levels were quantified and compiled into a unified transcripts set using Stringtie (v 1.2.4) (Pertea et al., 2015). Differential expression testing was performed using DESeq2 (Love et al., 2014). As the initial DESeq2 analysis produced a very large number of differentially expressed transcripts, we excluded any transcripts whose expression levels in any replicates were more than 25% greater or less than the mean expression level of all three replicates from the same tissue.

### 2.4 cDNA synthesis and qPCR analysis

One μg of pooled total RNA (equal amount of RNA from each plant) for each of the tissues was treated with TURBO DNase (Thermo Fisher Scientific, Waltham, MA, USA) to remove DNA molecules according to the manufacturer instruction. First strand cDNA synthesis was performed using SuperScript III cDNA synthesis system (Invitrogen, Carlsbad, CA, USA) in 20 μL volume containing 8 μL DNase treated RNA, 1 μL 50 μM oligo (dT), 1 μL 10 mM dNTP mix, 2 μL 10× RT buffer, 2 μL 0.1 M DTT, 1 μL RNaseOUT (40 U/μL), and 1 μL SuperScript III RT (200 U/μL) and 5 μL RNase‐free water. RNA, oligo (dT), dNTP mix, and water placed in 0.2 mL PCR tubes, incubated at 65°C for 5 min followed by 2 min hold on ice. Then RT buffer, DTT, RNaseOUT, and SuperScript III RT were added to the reactions and incubated at 50°C for 1 h, 75°C for 15 min, and hold on ice briefly. One μL of *Escherichia coli* RNase H (2 U/μL) was added to the reaction and incubated for 30 min at 37°C.

cDNA was then diluted to 20× with RNase‐free water. qPCR reactions were set up in 384‐well plate in a 10 μL volume containing 2 μL diluted cDNA, 1 μL forward primer (1 μM), 1 μL reverse primer (1 μM), 5 μL KiCqStart SYBR Green qPCR ReadyMix with ROX (Sigma‐Aldrich, St Louis, MO, USA), and 1 μL water. Primers for selected hairy vetch transcripts were designed using PrimerQuest Tool of IDT (Integrated DNA Technology, Coralville, IA, USA) with target amplicon size of 92–120 bp and melting temperature of 61.7–62.6°C. Primer sequences of hairy vetch target transcripts with annotations were presented in Table S7. cDNA from each of the tissues replicated three times along with no‐template controls. PCR reactions were run in QUANTStudio 7 (ABI, Waltham, MA, USA) using following PCR steps: (95°C 3 min) × 1 + (95°C 30 s, 60°C 1 min) × 40. Cycle threshold (CT) values were calculated using QUANTStudio software. Relative quantitations, delta–delta CT values, were calculated using formula: *R* = 2^−[∆^ *^CT^* ^(target)–∆^ *^CT^* ^(control)]^ (Pfaffl, 2004). HVRNA2945, a transcript annotated as *M. truncatula* tubulin beta‐1 chain gene, was used as control to estimate relative expression levels of target (samples) transcripts using formula described above. Average and standard deviations of relative expression over three replications were presented in bar graph created in R‐studio.

## 3 RESULTS

### 3.1 De novo assembly of RNA Seq data

Sequencing of RNA Seq libraries from multiple hairy vetch tissues collected from either hard or soft seeded plants produced 3122–7918 Mb sequence data (Table 1) for each of the tissue samples. The sample sequence variations occur most likely due to the technical errors during sequence library normalization and pooling. The RNA sequences translate into a 1040–2639× coverage per tissue based on a of the predicted hairy vetch genome size of 2000 Mb, with 1.5% of the plant genome containing genes. De novo super‐assembly of all the sequences followed by homology filtering yield 92,485 contigs with 73,227 Mb bases assembled in to the contigs (Table 2). Contig size ranged from 200 to 1651 bp with the average of 791.8 bp and N50 length of 871 bp. Mapping of the hairy vetch contigs against *M. truncatula* genome (V4.0) resulted in the identification of 76,422 predicted genes in hairy vetch. The rational for the use of *M. truncatula* genome is that it is a model legume species, and it's genome, genes, and transcriptome data are well characterized, well curated, and updated (Carrere et al., 2021; Garmier et al., 2017; He et al., 2009; Nandety et al., 2022).

**TABLE 1:** Hairy vetch RNA Seq data output from hairy vetch tissues with theoretical coverage.

Plant origin	Tissue	Reads (M)	Nucleotides (Mb)	Coverage (×)^a^
Soft seeded	Leaves	228.64	3430	1143
	Flowers	255.70	3836	1278
	Pods	214.55	3219	1073
	Seed coat	208.16	3122	1040
	Cotyledons	329.13	4938	1646
Hard seeded	Leaves	277.17	4157	1385
	Flowers	298.35	4480	1493
	Pods	229.10	3436	1145
	Seed coat	249.64	3745	1248
	Cotyledons	427.84	7918	2639

^a^ Assuming a 2000 Mb genome size of hairy vetch and ∼1.5% genome contains genes.

**TABLE 2:** Hairy vetch RNA sequence assembly statistics.

Total bases in contigs	73,227,779
Number of contigs	92,485
Minimum length (bp)	200
Maximum length (bp)	1651
N50 length (bp)	871
Mean length (bp)	791.8
Number of transcripts	76,422

### 3.2 Genes constitutively expressed in hairy vetch tissues

The fragments per kilobase of transcript per million mapped reads (FPKM) values equal to or greater than 10 and within replication variation of less than 10% (CV) after combining sequences from the same tissue types between hard and soft seeded plants were used to define genes expressed constitutively in different hairy vetch tissues. After removing duplicates, flower tissue showed the highest number of constitutively expressed genes (7361), followed by cotyledon (5265), pods (5147), and seed coat (3718) (Table 3). The lowest number of constitutively expressed genes was found in leaf tissues (2763). Details of the gene descriptions were presented in Table S1.

**TABLE 3:** Number of genes constitutively expressed in different hairy vetch tissues.

Leaves	Flowers	Pods	Seed coat	Cotyledons
2763	7361	5147	3718	5265

**FIGURE 1:**
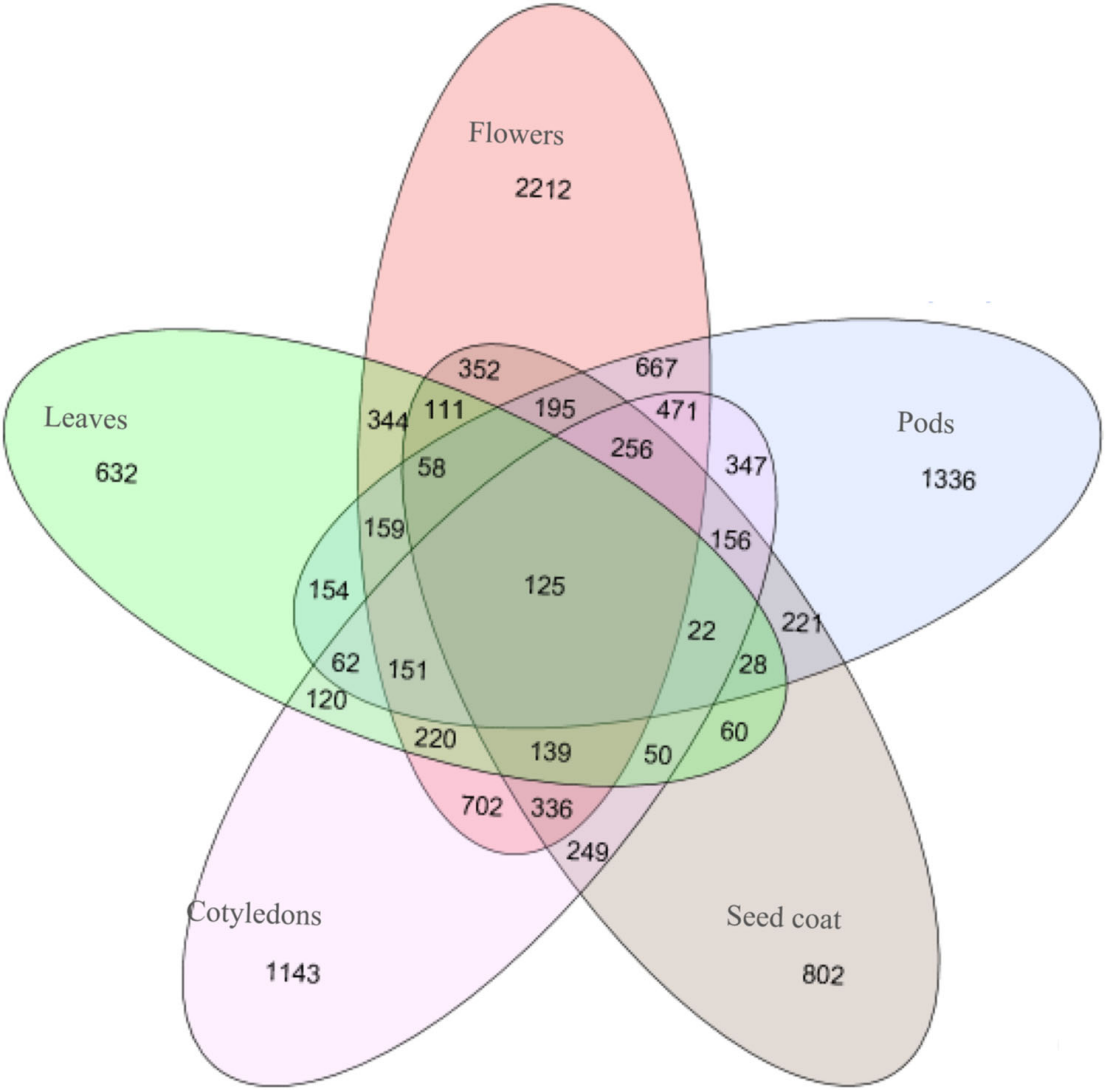
Venn diagram showing unique and shared set of constitutive expressed genes in different hairy vetch tissues.

**FIGURE 2:**
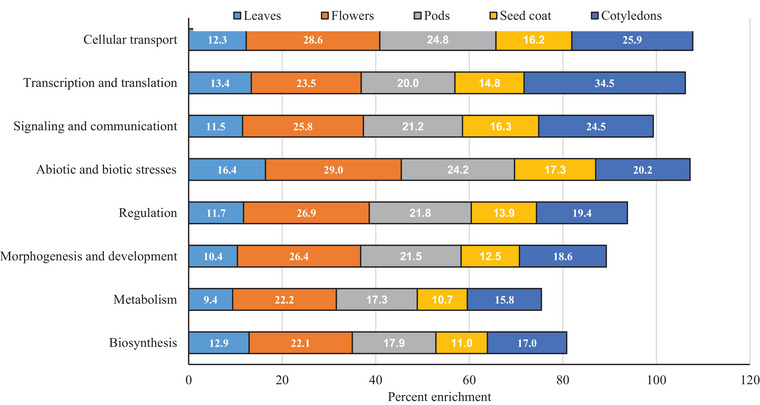
Significantly enriched gene ontology subcategories belonged to biological process function among different tissues of hairy vetch.

Venn diagram of the constitutively expressed genes showed that flowers (2212) possessed the highest number of unique set of genes followed by pods (1336) and cotyledons (1143) (Figure 1). Flowers shared 702 genes with cotyledons, 667 with pods, 352 with seed coats, and 344 with leaves. One hundred twenty‐five genes were shared by all the tissues. Gene ontology (GO) analysis of the annotated genes suggested that the genes constitutively expressed in leaves were enriched most (67.6%) in the hairy vetch tissue libraries followed by seed coat (57.4%) and cotyledons (54.2%) (Table 4) as compared to the *M. truncatula* transcripts. Detail analysis of significantly enriched genes belongs to biological process categories revealed that genes related to transcription and translation were highly enriched in the cotyledons (34.5%), whereas flower tissues showed the highest enrichment (29.0%) for stress response genes (Figure 2, Table S2). Leaves and seed coat also showed the highest enrichment for genes related to stress response as compared to other functional categories. In pods, genes involved in cellular transport were enriched most.

**TABLE 4:** Gene ontology (GO) enrichment of constitutively expressed genes in hairy vetch tissues.

Tissues	Annotated genes	GO defined	GO enriched (%)
Leaves	2433	1863	1260 (67.6)
Flowers	6498	4411	2018 (45.8)
Pods	4408	3480	1832 (52.6)
Seed coat	3160	2282	1309 (57.4)
Cotyledon	4549	3087	1672 (54.2)

### 3.3 Genes differentially expressed in hard seeded plant tissues

Differential gene expression analysis among different tissues of hard seeded plants revealed that the flowers showed the highest number of upregulated genes (2382) when compared with cotyledons (Table 5, Table S3). This was followed by flowers when compared with seed coat (1481). The flower tissue also showed the highest number of downregulated genes as compared to cotyledon (1622). Twenty and 15 genes were up‐ and downregulated, respectively, when seed coats were compared with cotyledons. Only three and two genes were up‐ and downregulated, respectively, when pods were compared with seed coat. Expression levels of differentially expressed genes varied from −51.4 to 59.4× in log2 fold scale (Table 5).

**TABLE 5:** Number of genes differentially expressed between tissues of hard seeded plants.

Tissue comparison		Downregulation	Upregulation	Total	Log2 fold change
Leaves	Flowers	290	720	1010	−30.0 to 23.7
	Pods	108	206	314	−50.9 to 7.0
	Seed coat	348	112	460	−52.0 to 59.4
	Cotyledons	1158	819	1977	−51.4 to 26.3
Flowers	Pods	367	958	1325	−21.9 to 26.9
	Seed coat	437	1481	1918	−23.6 to 24.1
	Cotyledons	1622	2382	4004	−27.0 to 26.4
Pods	Seed coat	2	3	5	−5.0 to 15.7
	Cotyledons	815	346	1161	−40.5 to 24.2
Seed coat	Cotyledons	15	20	35	−15.5 to 8.8

### 3.4 Genes differentially expressed in soft seeded plant tissues

Flowers showed the highest number of both up‐ (1224) and downregulated (1106) genes when comparisons were performed with cotyledons (Table 6, Table S4). This was followed by leaves when compared to the cotyledon‐ 968 up‐ and 984 downregulated. Ninety‐three and 85 genes were up‐ and downregulated, respectively, when seed coats were compared with cotyledons. Similar to the hard seeded tissue comparisons, only three genes were upregulated and three genes were downregulated when pods were compared with seed coat. Expression levels of differentially expressed genes varied from −38.6 to 41.4× in log2 fold scale (Table 6).

**TABLE 6:** Number of genes differentially expressed between tissues of soft seeded plants.

Tissue comparison		Downregulation	Upregulation	Total	Log2 fold change
Leaves	Flowers	235	340	575	−11.8 to 34.7
	Pods	12	25	37	−6.32 to 11.7
	Seed coat	261	310	571	−10.6 to 24.4
	Cotyledons	984	968	1952	−35.6 to 24.0
Flowers	Pods	46	223	269	−7.8 to 26.4
	Seed coat	280	490	770	−38.6 to 24.7
	Cotyledons	1106	1224	2330	−38.2 to 41.4
Pods	Seed coat	3	3	6	−4.8 to 4.8
	Cotyledons	153	262	415	−16.0 to 24.3
Seed coat	Cotyledons	85	93	178	−6.5 to 22.6

### 3.5 Genes differentially expressed between hard and soft seeded plant tissues

**FIGURE 3:**
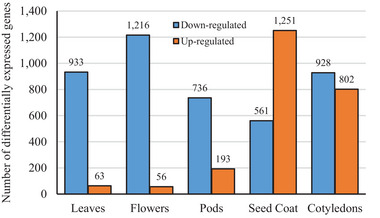
Bar graph showing differentially expressed genes between hard and soft seeded tissues.

Differential gene expression analysis between the same tissue derived from hard and soft seeded plants revealed that the highest number of genes upregulated in seed coat (1251) (Figure 3). This was followed by cotyledons. Flower tissue exhibited the highest number of downregulated genes (1216) followed by leaves (933) and cotyledons (928). More genes downregulated in pods than upregulated. Expression level of these genes varied from −51.3 to 46.9 (log2 values) (Table S5).

### 3.6 Unique and shared set of differentially expressed genes in hard versus soft tissues

**FIGURE 4:**
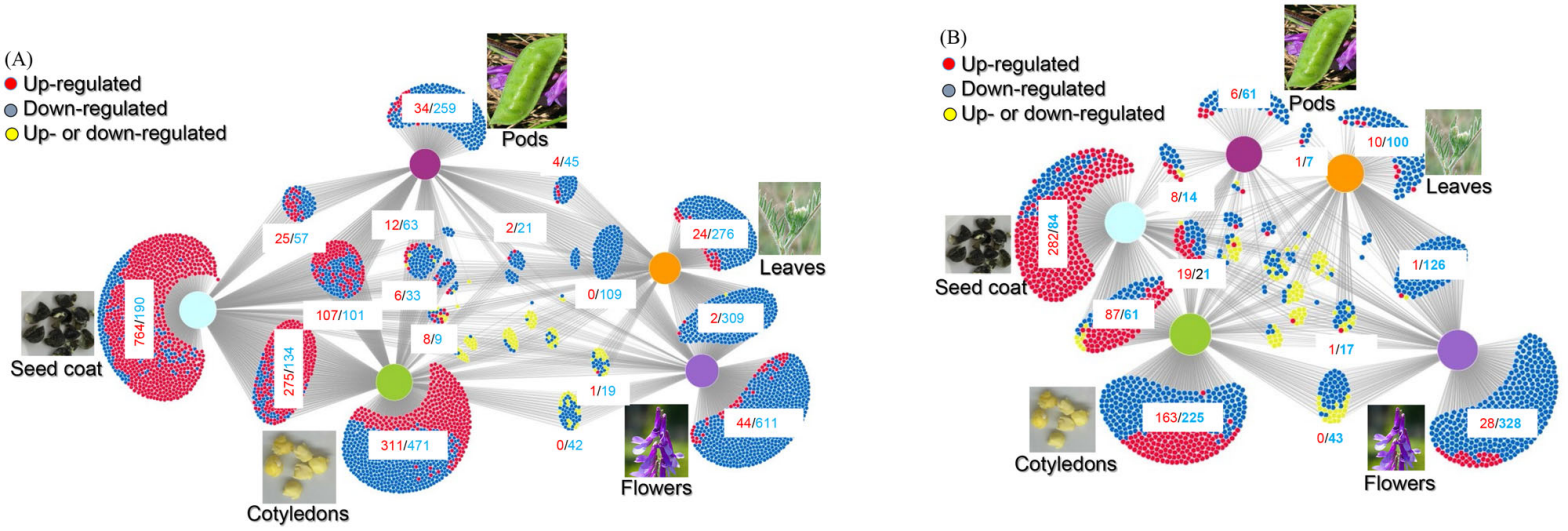
Venn diagram showing unique and shared set of differentially expressed genes in multiple tissues of hairy vetch: unique transcripts (A) and annotated unique transcripts (B). A number of genes that are up‐ and downregulated are displayed in the figures as red and blue text colors, respectively.

To identify unique and shared set of differentially expressed transcripts among tissues of hard versus soft seeded plants, we created a Venn diagram using the nonredundant set of transcripts in DiVenn software (Sun et al., 2019). Number of genes uniquely expressed in seed coat, cotyledons, and pods were 954, 782, and 239, respectively. We observed a large number of transcripts uniquely upregulated (764) than downregulated (190) especially in seed coat (Figure 4A). Seed coat and cotyledon shared 409 transcripts (275 upregulated vs. 134 downregulated), whereas only 82 and 85 genes were common between seed coat and pods, and cotyledon and pods, respectively. All of these 3 tissues shared 208 genes (107 up‐ and 101 downregulated). Leaves and flowers possessed 313 and 655 unique genes, respectively, but most of them were downregulated. As many transcripts (43.8%–78.6%) were unannotated (no hit in blast search against *M. truncatula* data base) while analyzing hairy vetch transcript libraries, we recreated the Venn diagram using annotated sets of genes expressed (Figure 4B). We observed more upregulated (292) than downregulated (64) genes in the unique set of seed coat. Seed coat shared 97 and 51 up‐ and downregulated genes, respectively, with cotyledon. All of the other tissues possessed more downregulated genes as compared to the upregulated ones in their unique sets as well as shared set of genes.

### 3.7 Gene ontology enrichment of differentially expressed genes in hard seed coat

**FIGURE 5:**
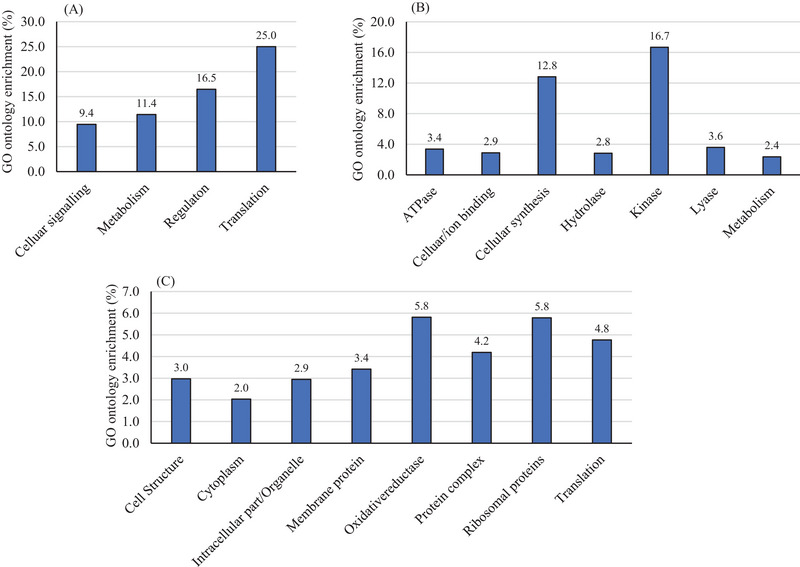
Significantly enriched gene ontology (GO) subcategories for differentially expressed genes in seed coat belonged to biological process (A), molecular function (B), and cellular components (C).

GO analysis of 704 annotated genes that were expressed differentially in hard seed coat revealed that overall, 3.1% of the genes were significantly enriched in all three major functional categories: biological process, molecular function, and cellular components (Table S6). Among the biological process functional category, genes involved in translation were most enriched (25%) followed by genes involved in regulation (16.5%) (Figure 5A). In the molecular functional category, kinases genes were enriched most (16.7%) followed by genes involved in cellar synthesis (12.8%) (Figure 5B). Genes involved in oxidoreductase (5.8%), ribosomal protein (5.8%), translation (4.8%), and protein complexes (4.2%) were enriched most in the cellular component functional category (Figure 5C).

### 3.8 Expression of genes relevant to seed hardness and other traits

We have identified a number of gene homeologs in hairy vetch transcriptome libraries after looking through annotated genes and homology searching (Table 7). These genes are involved in seed hardness, seed shattering, flowering, photosynthesis, and plant growth and development. A blast search result showed more than one transcript aligned with each of the genes with various degrees of identity. We listed the transcripts with 84%–97% nucleotide identity with read length of 231–4105 bp (Table 7). These genes were constitutively expressed in one or more tissues and/or expressed differentially.

**TABLE 7:** List of genes relevant to seed hardness and related traits.

Hairy vetch transcript ID	Gene description	Nucleotide identity % (length bp)	Tissue(s) expressed (FPKM ≥ 10)
HVRNA.45679^a^	*Pisum sativum* class II knotted‐1‐like 4 homeobox transcription factor	95 (588)	SF, SP, HF, HP, HS
HVRNA.61680^a^	*Cicer arietinum* endoglucanase 11‐like protein*/GmqHs1*: soybean endo‐1,4‐β‐glucanase	86 (428)	SS, HS
HVRNA.64776^a^	GmHs1‐1: soybean calcineurin‐like metallo‐phosphoesterase	84 (518)	SL, SF, SS, SC, HL, HS, HC
HVRNA.37118^a^	*Medicago truncatula* expansin‐A17/soybean expansin A like protein	84 (602)	SP, HP, HS
HVRNA.80905	SHP1: soybean shatter proof gene 1	92.0 (2046)	NA
HVRNA.33465	*C. arietinum* PHYTOCHROME‐DEPENDENT LATE‐FLOWERING protein/SHP2: soybean shatter proof gene 2	85 (3780)	SP, HL, HF, HS, HC
HVRNA.55793	*C. arietinum* 3‐oxoacyl‐[acyl‐carrier‐protein] synthase I/soybean Pdh1	89 (1433)	SF, SC, HF, HS, HC
HVRNA.17076	SHAT1	92.3 (4505)	NA
HVRNA.83408^a^	*M. truncatula* ABC transporter G family member 28/soybean IND	91 (2672)	SF, HS
HVRNA.20800	*M. truncatula* uncharacterized protein/soybean chitinase II	93 (1284)	SF, HF
HVRNA.68602^a^	*M. truncatula* cellulose synthase‐like protein H1/soybean cellulose synthase II	89 (1778)	SC
HVRNA.55962	SHAT1–5	95.0 (1744)	SP
HVRNA.77116	*M. truncatula* uncharacterized/soybean SHAT1–5	88 (1643)	SP, SS, HF, HS
HVRNA.3671	*C. arietinum* MACPF domain‐containing protein/soybean, common vetch INDI	85 (512)	HF
HVRNA.24041^a^	*M. truncatula* 21 kDa protein/soybean, common vetch Pectinoisomerase	87 (586)	SF
HVRNA.45344	Common vetch Endo‐B1‐3 glucosidase I	93 (1481)	NA
HVRNA.82887^a^	*P. sativum* β‐galactosidase‐like protein/common vetch β‐galactosidase	93 (1481)	SL, SF, SP, HL, HF, HP, HS
HVRNA.79031^a^	*M. truncatula* cellulose synthase‐like protein H1/common vetch cellulose synthase I	85 (1279)	SL, SC, HL, HC
HVRNA.36815^a^	*M. truncatula* expansin‐A10/common vetch expansin	84 (621)	SL, SF, SP, HF, HP
HVRNA.6742	B glucosidase I	95.2 (945)	SL, SF, SP, HL, HF, HP
HVRNA.59301^a^	Vigna angularis carboxymethylenebutenolidase homolog/common vetch endo‐β‐1‐3 1‐4 glucanase	85 (231)	SL, HL
HVRNA.50883^a^	*M. truncatula* scarecrow‐like protein 6/grass family transcription factor/regulator	87 (1950)	NA
HVRNA.68181^a^			
	*C. arietinum* EARLY‐RESPONSIVE TO DEHYDRATION protein7		
	91 (730)	SL, SF, SP, SS, HL, HF, HP, HS	
HVRNA.70473^a^	*M. truncatula* senescence‐associated protein	88 (1242)	SL, SF, SP, HL, HF HP, HS
HVRNA.40268^a^			
	*M. truncatula* floral homeotic protein APETALA 2		
	83 (573)	SL, SF, SS, HL, HF,	
HVRNA.57565	*M. truncatula* ethylene‐responsive transcription factor RAP2–7	86 (673)	SL, SF, SP, HL, HF, HP
HVRNA.50009^a^			
	*M. truncatula* cinnamoyl‐CoA reductase 2		
	88 (297)	SL, SF, SP, SS, SC, HL, HF, HP, HS, HC	
HVRNA.20352^a^	*M. truncatula* cinnamyl alcohol dehydrogenase‐like protein	88 (908)	SL, SF, SP, HL, HF, HP
HVRNA.73517^a^			
	*P. sativum* MADS‐box transcription factor NMH7 *M. truncatula* agamous‐like MADS‐box protein AP3		
	97 (405)	SF, HF	
HVRNA.58776^a^	*P. sativum* mRNA for stay green like (SGRL gene)	96 (630)	SL, SF, SP, HL, HF, HP
			
HVRNA.49351^a^	*M. truncatula* NAC domain‐containing protein		
	86 (1013)	SF, SP, SS, HF, HP, HS	
HVRNA.38943^a^	*M. truncatula* NAC domain‐containing protein 43	89 (1482)	SF, HP
HVRNA.82777^a^			
	*P. sativum* early flowering protein		
	91 (1090)	SL, SS, SC, HF, HS, HC	
HVRNA.80435^a^			
	*C. arietinum* nucleolar protein 10/*M. truncatula* embryo sac development arrest protein		
	91 (935)	SF, SS, SC, HF, HS, HC	
HVRNA.55603^a^	*M. truncatula* EMBRYONIC FLOWER 1 protein	88 (2024)	SF, SS, SC, HF, HS, HC
HVRNA.53286	*M. truncatula* transmembrane emp24/gp25L/p24 family protein	91 (523)	SL, SF, SP, SS, SC, HL, HF, HP, HS, HC
HVRNA.77784^a^			
			
	*C. arietinum* CSC1‐like protein/ERD (early‐responsive to dehydration stress) family protein		
	91 (2267)	SF, SS, HF, HS	
HVRNA.75729^a^	*M. truncatula* angio‐associated migratory cell protein/vegetative incompatibility HET‐E‐like protein	89 (671)	SL, SF, SS, SC, HL, HP, HS, HC
HVRNA.52321^a^	*M. truncatula* probable pectate lyase 8/common vetch pectate lyase	89 (770)	SF, SP, HF, HP
HVRNA.53582^a^	*M. truncatula* glucuronokinase 1	89 (961)	SF, SP, SS, HF, HP, HS
HVRNA.80614^a^	*M. truncatula* phosphatidylinositol‐glycan biosynthesis class F protein/common vetch Chitinase II	89 (546)	SP, HP, HS
HVRNA.61477^a^	*C. arietinum* eukaryotic peptide chain release factor GTP‐binding subunit/elongation factor Tu (EF‐Tu) GTP‐binding family protein	90 (1.179)	SL, SF, SP, SS, SC, HL, HF, HP, HS, HC
HVRNA.62480^a^	*C. arietinum* arogenate dehydrogenase 1/soybean Pdh1	88 (600)	SF, SS, HF, HS
HVRNA.38943	*M. truncatula* NAC domain‐containing protein 43/soybean SHAT1 (NAC transcription factor)	89 (1482)	SP, HP
HVRNA.9458^a^	SHP2: *C. arietinum* agamous‐like MADS‐box protein AGL11	87 (708)	SP, SS, HP, HS
HVRNA.52688^a^	*M. truncatula* NAC domain‐containing protein 7/soybean SHAT1–5	84 (664)	NA

*Note*: NA, gene not found in any hairy vetch tissues.

Abbreviation: FPKM, fragments per kilobase of transcript per million; HC, hard cotyledon; HF, hard flower; HL, hard leaf; HP, hard pod; HS, hard seed coat; SC, soft cotyledon; SF, soft flower; SL, soft leaf; SP, soft pod; SS, soft seed coat.

^a^ qPCR analysis performed.

### 3.9 qPCR analysis of representative genes

**FIGURE 6:**
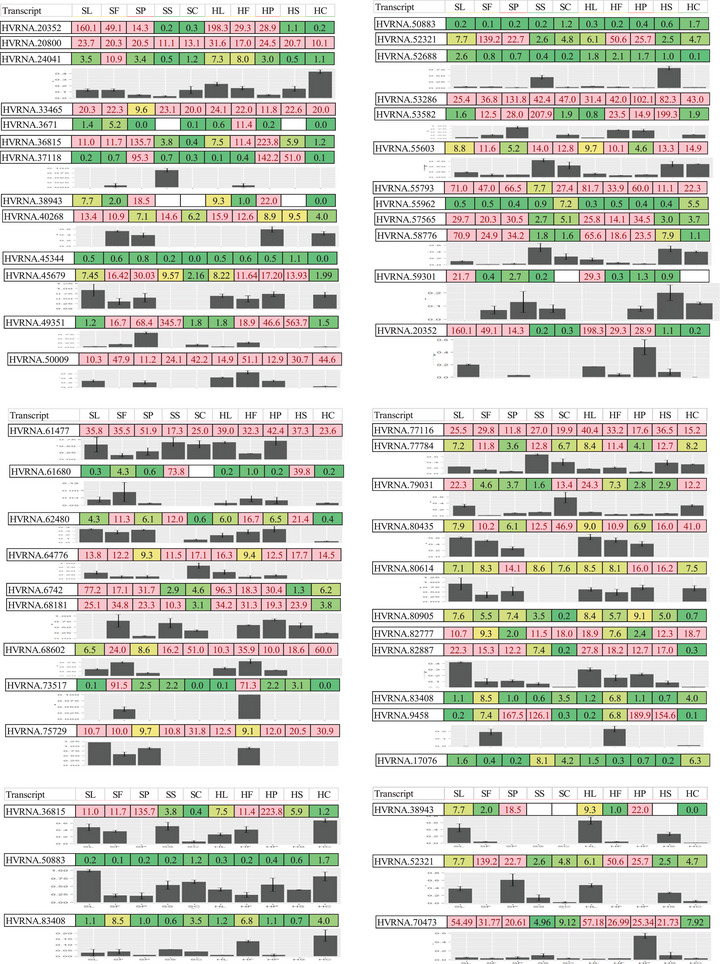
Color coded fragments per kilobase of transcript per million (FPKM) values of the genes—light red (equal to or greater than 10.0), yellow (6.8–9.7), and green (equal to or less than 6.1) and their expression level in qPCR. HC, hard cotyledon; HF, hard flower; HL, hard leaf; HP, hard pod; HS, hard seed coat; SC, soft cotyledon; SF, soft flower; SL, soft leaf; SP, soft pod; SS, soft seed coat.

We assayed 35 transcripts in qPCR, including 3 housekeeping genes, namely, *Vicia faba* actin (HVRNA.34775), *M. truncatula* tubulin β‐1 chain (HVRNA2945), and (HVRNA.61477) *Cicer arietinum*/elongation factor Tu. Among these, HVRNA2945 produced robust CT values across all of the tissues and hence used for relative expression analysis. Delta–delta CT values were calculated for 320 assays (32 transcripts × 10 tissue combinations). Overall, 72.8% (233 of 320 assay set) qPCR assays were successful in producing expression data. Relative expression of 19.7% (63 of 320) assay could not be estimated because the CT values of HK genes were lower than the target transcripts with only 7.5% (24 of 320) of the qPCR assay failure. We presented qPCR results of 32 transcripts with FPKM values at the top of each of the transcripts (Figure 6). qPCR expression results of the 62.5% (20 of the 32 transcripts) transcripts agree with the FPKM values at most of the tissues, examples are RNA.55603 (*M. truncatula* EMBRYONIC FLOWER 1 protein), RNA.64776 (Gm*Hs1‐1*: Soybean calcineurin‐like metallo‐phosphoesterase), RNA.68181 (*C. arietinum* EARLY‐RESPONSIVE TO DEHYDRATION protein7), RNA.79031 (*M. truncatula* cellulose synthase‐like protein H1/Common vetch Cellulose synthase I), and RNA.82887 (*P. sativum* early flowering protein).

## 4 DISCUSSION

In our ongoing efforts of generating genomic resources for hairy vetch, a de novo species, transcriptome profiling was performed using RNA Seq technology with the aspiration of cataloging a complete set of genes expressed constitutively in different tissues and to identify genes differentially expressed especially in seed coats using a BSA method. BSA coupled with RNA sequencing (called BSR‐seq) is a recent approach to identify differentially expressed genes in crop species and to identify contrasting markers between two pools (Du et al., 2017; Edae & Rouse, 2019; Liu et al., 2019; Nishijima et al., 2018). To create a resourceful transcriptome profiling with the presence of low abundant ones, requires RNA sequencing with enough coverage depth as abundance of mRNA varies from 1 to 50,000 copies per cell depending on species and cell type (Carninci et al., 2000; Goldberg et al., 1978). Coding sequences of plant genomes typically represent only 1%–2% of a genome (Warr et al., 2015) and comprise high level of functional variants and low repeat content. In wheat, exome constitutes 170–340 Mb (Paux et al., 2006) of size as compared to massively large genomes of tetraploid (10,000 Mb) and hexaploid (17,000 Mb) wheat (Kaur & Gaikwad, 2017). Although hairy vetch has genome size of 2000 Mb, it contains ∼81% of repeat sequences (Vvil1.0 draft assembly, not published). The coverage of the RNA sequencing data of hairy vetch transcript libraries varied from 1043 to 2639× with the conservative estimation 1.5% of coding regions.

In identifying genes that were expressed constitutively in different tissues, very stringent filtering criteria with the FPKM value of at least 10 were adapted in this study. Most of the transcriptome analysis of plant species FPKM value equal or greater than three used to delineate genes expressed in specific tissues (O'Rourke et al., 2014). Expression of large number of genes in the flower tissues in this study was not unexpected because of the transition from vegetative to reproductive stage and plants preparedness for pods and seed development and maturation. In transcriptome analysis of common bean, O'Rourke et al. (2014) also reported highest number of genes expressed in flowers as compared to leaf, pods, and seeds. We have identified a number of well‐characterized genes that are expressed in specific tissues in vetch. Functions of the some of the genes are described in following paragraphs. Despite the bottlenecks in isolating RNA from pods, seed coat, and cotyledons without confirming whether they were derived from soft or hard seeds, identification of number of well‐characterized genes expressed in different tissues coupled with qPCR expression agreement with the FPKM values of more than 72% of the transcripts increased our confidence level of the reliability of thisstudy. Since our study was completed, more closely related genomomes have been published (Xi et al., 2022), which would improve the identification of transcript for *Vicia villosa* in the future.

Differentially expressed gene analysis revealed that cotyledon tissues always produced higher number of up‐ or downregulated genes when compared with the other tissues irrespective of hard or soft tissue source. Grain filling or endosperm development in legumes is the longest biological processing stage. In a gene expression study in seed development in common bean, O'Rourke et al. (2014) reported that 50% of the 31,638 predicted genes were expressed in the 3 stages of seed development. The authors described three seed development stages as: heart stage seeds when seeds are 3 and 4 mm across and ∼7 mg in weight; Stage 1 seeds when seeds are 6 and 7 mm across and ∼50 mg weight; and Stage 2 seeds when seeds were 8 and 10 mm across and 140–150 mg weight. Identification of only few differentially expressed genes in seed coat as compared to the pods implied that genes involved in seed coat development and maturity started to express at the early stage of pod/seed development. Although seed coat tissues were isolated at physiological matured seeds indicated by dark brown color of the seeds, significant enrichment of genes involved in translation, ribosomal protein, protein complexes, kinases, regulation, and biosynthesis indicated that seed coats were still functionally active when seeds were harvested for this study. We also observed that 52.9% of the upregulated transcripts in the seed coat of hard tissue were not annotated. Differential expression levels (log2 fold change) of some of the transcripts were very high (up to 33.9). It might be possible that these transcripts are unique in hairy vetch seed coat tissues. In legumes, only a limited number of studies have been published on the gene expression in the seed coats. In a differential gene expression study in cultivated versus wild‐type pea seed coats using massive amplification of cDNA ends (MACE), Hradilová et al. (2017) could annotate only 66% of the MACE fragments because of the absence of the complete pea genome and specificity of seed coat tissues. Moreover, authors reported that 36%–41% transcripts were assignment ambiguously. Kour et al. (2014) reported differential gene expression studies between wild‐type and net pattern mutant of soybean seed coat and observed that the abundances of the transcripts related to proline rich proteins, growth hormones, and transcription factors were reduced in the mutant lines.

We have identified the number of genes involved in seed hardness, pod shattering, and other important phenotypic traits. They are expressed constitutively and/or expressed differentially. KNOX4, a class II KNOTTED‐like homeobox (*KNOXII)* gene, and its target gene, *CYP86A*, was involved in seed hardness. TN1 mutant of KNOX4 in *M. truncatula* showed cracks in the palisade tissues and resulted into permeable seeds (Chai et al., 2016). The *KNOX4* homologs in hairy vetch were expressed all of the tissues except leaves and soft cotyledon. This transcription factor gene is the target for members of cytochrome P450 monooxygenase, *CYP86A*, which is associated with extracellular lipid polyester biosynthesis in particular *CYP86A8* that is involved in cutin biosynthesis in *Arabidopsis thaliana* (Wellesen et al., 2001). A homolog of soybean *qHs1* (endo‐1,4‐β‐glucanase) was expressed in the seed coat of hairy vetch, whereas *GmHs1‐1* (calcineurin‐like metallo‐phosphoesterase) expressed all of the hairy vetch tissues. The hard seed allele of *qHs1* involved in the accumulation of glucan derivatives at the point of water entry at the outer layer of palisade cells of the seed coat located on the dorsal side of seeds (Jang et al., 2015). An introduction of SNP changed amino acid at the substrate binding side of the enzyme resulted in permeable seeds. Gm*Hs1‐1* encoded a calcineurin‐like metallophosphoesterase transmembrane protein that controls seed‐coat permeability in soybean (Sun et al., 2015). It is expressed in malpighian layer of the seed coat and is associated with calcium content. Mutant phenotype of Gm*Hs1‐1* showed seed‐coat cracking and enabled water permeability.

The major phenomenon of seed shattering is the alteration in cell structures at the dehiscence zone especially at the ventral suture of pods (Dong et al., 2014, 2017). A number of cell wall modifying enzymes and genes involved in cell structures are associated in this process, including endo‐polygalacturonase, pectin methylesterases, pectate lyase, β‐galactosidase (Christiansen et al., 2002; Dong et al., 2017; Tavarini et al., 2009; Zhang et al., 2012), glucosidase (Dong et al., 2017; Muriira et al., 2015), endo‐glucanase (Dong et al., 2017), SHATTERPROOF1 and 2 (*SHP1, SHP2*) (Liljegren et al., 2000), SHAT1–5/NAC transcription factor (Dong et al., 2014), and pdh1 (Funatsuki et al., 2014). Soybean and common vetch homologs of pod shattering were found expressed in the hairy vetch RNA Seq libraries. A number of them expressed in tissue‐specific manner, whereas some of them expressed in all of the tissues analyzed. Although Dong et al. (2017) did not observe differential expression of soybean and *Arabidopsis SHAT1–5*, *pdh1, SHP1, SHP2, INDEHISCENT (IND)*, and *ALCATRAZ (ALC)* genes in shatter resistant and susceptible genotypes, expression of some of these genes particularly in pods and seed coats opens the gateway to characterize their association with pod shattering in hairy vetch.

STAY‐GREEN (*SGR*) gene encodes a protein localized in chloroplast that dissociates photosynthetic chlorophyll–apoprotein complexes during senescence process in plants (Hörtensteiner, 2009). Leaf senescence is series of programmed degradative processes leading to the reallocation of nutrients to young leaves, seeds or stored organs, and eventual leaf death. Plant age as well as different stresses leads to the activation of the senescence process (Li et al., 2013; Liang et al., 2014; Seo et al., 2011). Manipulating leaf senescence through breeding or genetic engineering helps in crop yield improvement by keeping leaves photosynthetically active for longer period (Quirino et al., 2000; Rivero et al., 2007). Mutant lines of SGR genes increased crop yield by delaying the senescence mechanism (Thomas & Howarth, 2000; Yoo et al., 2007) or through cytokinin mediated increased drought resistance (Rivero et al., 2007). *SRG* gene homologs of hairy vetch (HVRNA.58776) expressed in leaves, flowers, and pods in this study and also upregulated differentially in soft flowers when compared to seed coat and cotyledons. Muchero et al. (2013) identified QTLs for stay‐green like traits that improved cowpea biomass and grain yield in drought stress conditions by delaying senescence.

APETALA2 (*AP2*) homologs of hairy vetch expressed in hard flower and seed coat. This gerne is a negative regulator of *SHP1, SHP2*, and *RPL*, and it inhibits the overexpression of these genes in the replum and valve margin of dehiscence zone of *A. thaliana* siliques (Ripoll et al., 2011). In a comparative, expression analysis of genes involved in fruit shattering of *Lepidium campestre* having dehiscent fruits with indehiscent *Lepidium appelianum* fruits, indicating that *AP2* involved in the regulation of these shattering genes (Mühlhausen et al., 2013). It was also revealed that stronger expression of *AP2* in indehiscent fruits as compared to the dehiscent fruits.

## 5 CONCLUSIONS

Results of this study postulates that hairy vetch RNA libraries are very resourceful in finding homologs of the genes involved in important phenotypic traits. These genes might be target for manipulations through advanced technology such as genome editing for the development of novel germplasm for hairy vetch cultivar development. As hairy vetch is a de novo species and genome assembly is currently in progress, RNA Seq data of the transcript libraries will help in the extent of the genome assembly and annotation. It can also be valuable in variant calling as a reference sequence in the absence of the draft/published genome. Moreover, plants generated from hard or soft seeds have practical implications in breeding hairy vetch. By inter‐mating plants generated from hard seeds, we generated hairy vetch population with net increase of 22% to 47% hard seeds in one generation. This might be true for generating soft seeded populations but needs to be tested.

## AUTHOR CONTRIBUTIONS

**Shahjahan Ali**: Conceptualization; Data curation; Formal analysis; Funding acquisition; Methodology; Validation; Visualization; Writing–original draft; Writing–review & editing; **Lisa Kissing Kucek**: Conceptualization; Writing–review & editing; **Heathcliffe Riday**: Conceptualization; Project administration; Writing–review & editing; **Nick Krom**: Data curation; Formal analysis; Software; Validation; **Sarah Krogman**: Resources; Validation; **Kimberly Cooper**: Resources; Validation; **Lynne Jacobs**: Resources; Validation; **Perdeep Mehta**: Data curation; Formal analysis; Resources; Software; Visualization; **Michael Trammell**: Resources; Writing–review & editing; **Suresh Bhamidimarri**: Conceptualization; Methodology; Supervision; Writing–review & editing; **Twain Butler**: Funding acquisition; Project administration; **Malay C. Saha**: Conceptualization; Supervision; Writing‐review & editing; **Maria J. Monteros**: Conceptualization; Supervision; Writing–review & editing.

## CONFLICT OF INTEREST STATEMENT

The authors “Shahjahan Ali, Nick Krom, Sarah Krogman, Kimberly Cooper, Lynne Jacobs, Perdeep Mehta, Michael Trammell, Suresh Bhamidimarri, Twain Butler, Malay C. Saha, and Maria J. Monteros” were employed by Noble Research Institute, LLC, Ardmore, OK, USA. The remaining authors declare that the research was conducted in the absence of any commercial or financial relationships that could be construed as a potential conflict of interest.

## GENE BANK SUBMISSION

RNA sequencing data generated for this research have been submitted to NCBI (April 28, 2022). The BioProject submission reference ID number is: PRJNA833581.

## Supporting information

Table S1 Genes expressed constitutively in hairy vetch tissues.
Table S2 Significantly enriched constitutively expressed genes belong to biological process in hairy vetch libraries.
Table S3 Genes differentially expressed between hard seeded tissues.
Table S4 Genes differentially expressed between soft seeded tissues.
Table S5 Genes differentially expressed genes between hard and soft seeded tissues.
Table S6 Significantly enriched differentially expressed genes in seed coats.
Table S7 qPCR primer sequences.
